# Supplementary figures and images for: Mutations in nucleotide metabolism genes bypass proteasome defects in png-1/NGLY1-deficient Caenorhabditis elegans
Source: PLoS Biol. 2024 Jul 11;22(7):e3002720. doi: 10.1371/journal.pbio.3002720 (PMC11265709; doi:10.1371/journal.pbio.3002720)

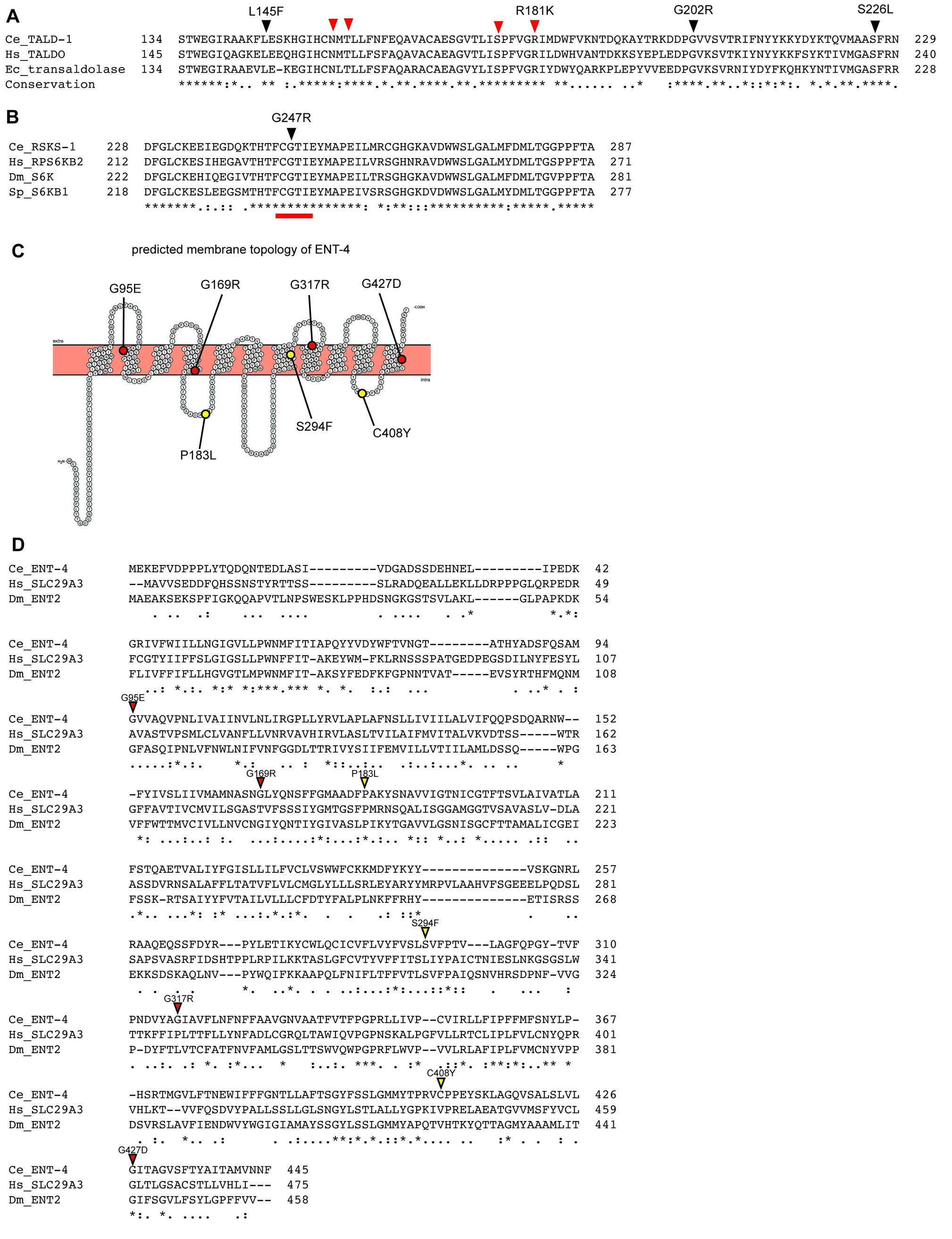

Supplement: S1 Fig — (A) Multiple sequence alignment of C. elegans TALD-1 (residues 134–229) with human and E. coli orthologs. Missense mutations isolated in the png-1Δ suppressor screen are labeled. Red arrows indicate conserved residues that form the transaldolase enzyme active site. (B) Multiple sequence alignment of C. elegans RSKS-1 (residues 228–287) with human, D. melanogaster and Strongylocentrotus purpuratus orthologs. The effect of the missense mutation isolated in the png-1Δ suppressor screen is labeled. The red underline marks conserved residues of the kinase active site. (C) Predicted membrane topology of ENT-4. Locations of missense mutations isolated in the png-1Δ suppressor screen are highlighted. Locations of amino acid substitutions that introduce a non-polar residue into a putative transmembrane helix are highlighted in red. Locations of other amino acid substitutions are highlighted in yellow. (D) Multiple sequence alignment of C. elegans ENT-4 with human SLC29A3 and Drosophila Ent2. Missense mutations isolated in the png-1Δ suppressor screen are labeled. (TIF) [file pbio.3002720.s001.tif]

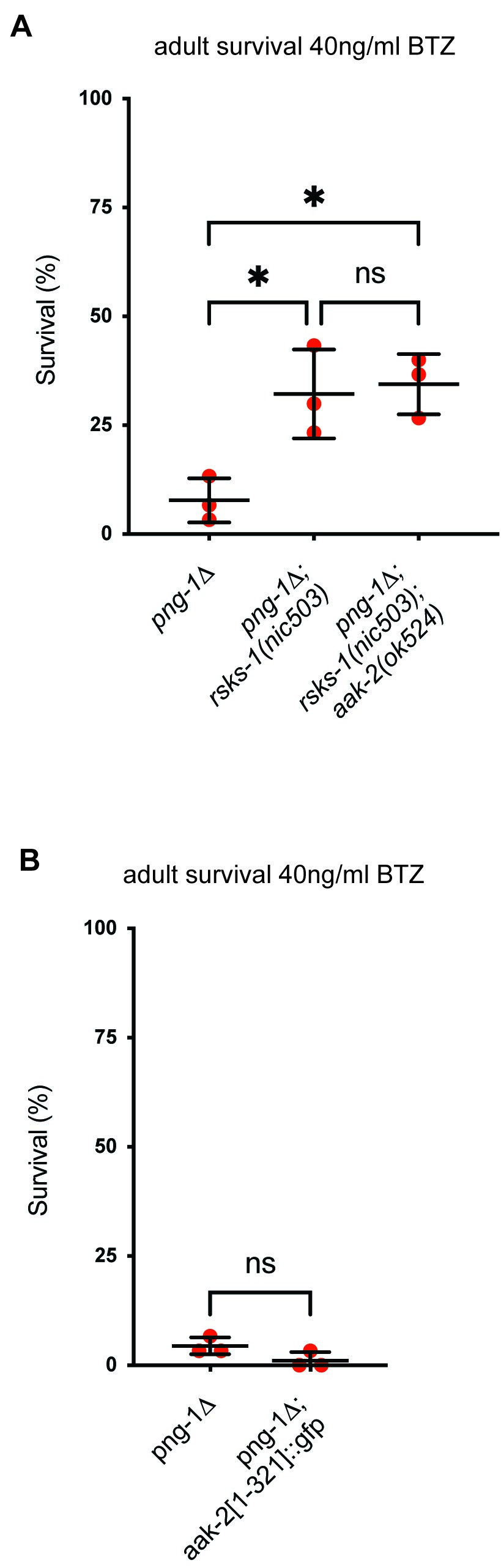

Supplement: S2 Fig — (A) Survival of adult animals exposed to 40 ng/ml bortezomib. Late L4 stage animals were shifted to bortezomib-supplemented plates and checked for survival after 4 days. The increased survival of png-1Δ conferred by rsks-1(nic503) does not require AAK-2. Results of n = 3 replicate experiments are shown; error bars show mean ± SD. Survival of 30 animals was tested for each replicate experiment. * p < 0.05, ns p > 0.05 (ordinary one-way ANOVA with Tukey’s multiple comparisons test). (B) Survival of adult animals exposed to 40 ng/ml bortezomib. Late L4 stage animals were shifted to bortezomib-supplemented plates and checked for survival after 4 days. The survival of png-1Δ animals is not improved by hyperactivation of AAK-2. Results of n = 3 replicate experiments are shown; error bars show mean ± SD. Survival of 30 animals was tested for each replicate experiment. ns p > 0.05 (Unpaired t test). Numerical data for both panels is available in S1 Data. (TIF) [file pbio.3002720.s002.tif]

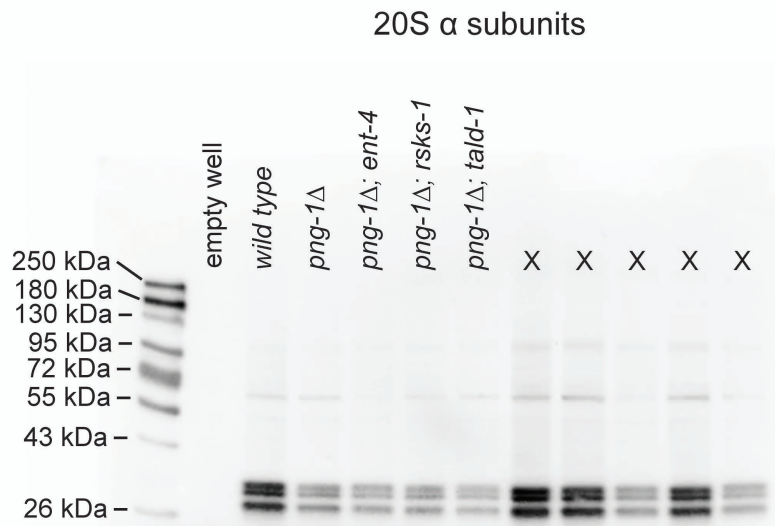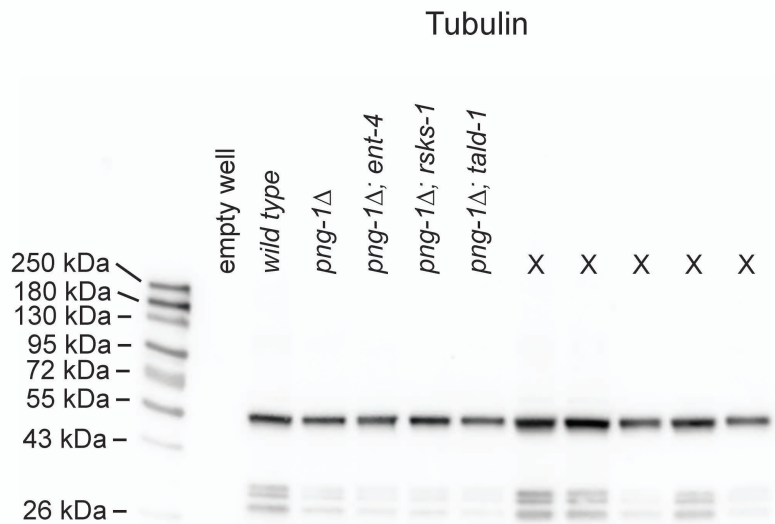

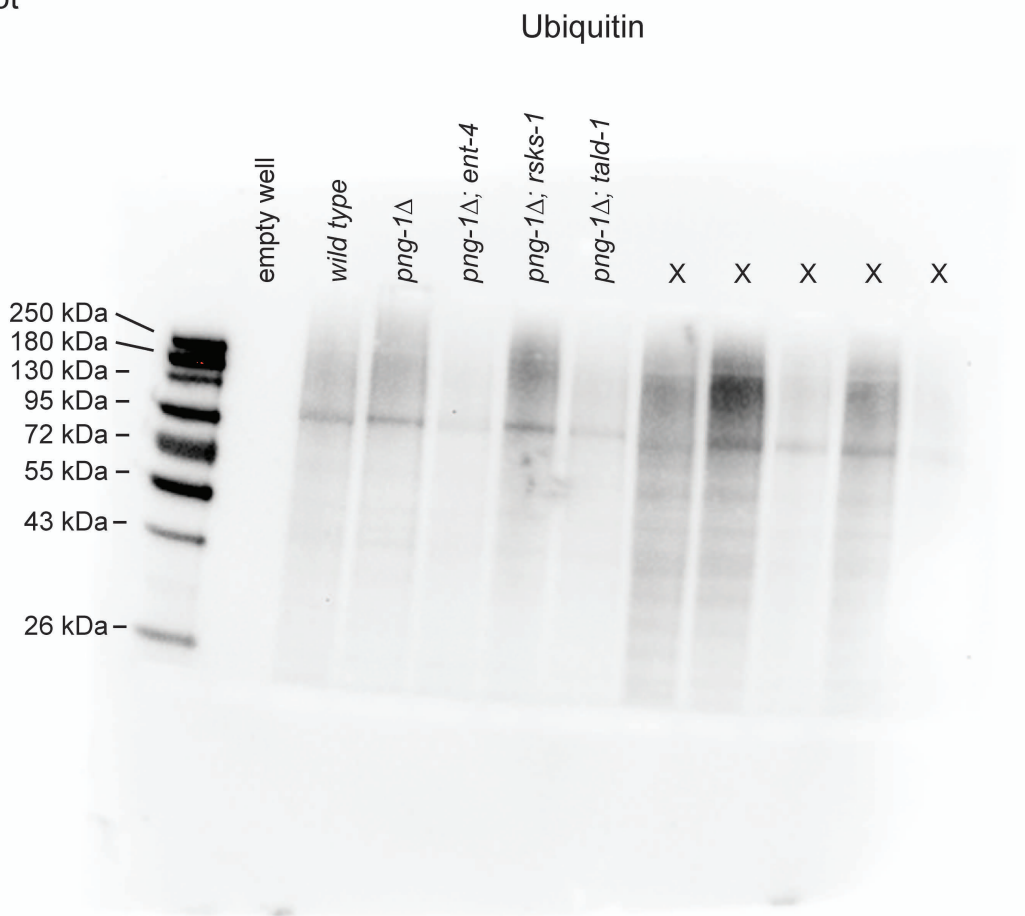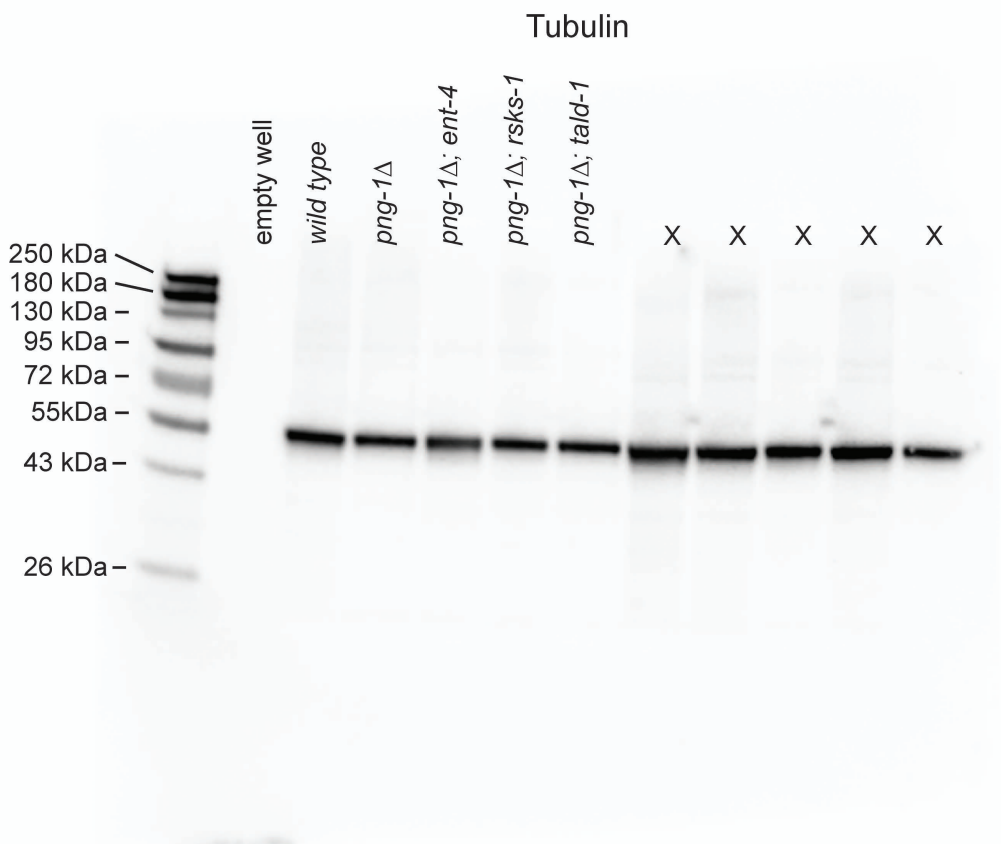

Supplement: S1 Raw Images — (PDF) [file pbio.3002720.s005.pdf]
