# Supplementary material for: Mutations in nucleotide metabolism genes bypass proteasome defects in png-1/NGLY1-deficient Caenorhabditis elegans
Source: PLoS Biol. 2024 Jul 11;22(7):e3002720. doi: 10.1371/journal.pbio.3002720 (PMC11265709; doi:10.1371/journal.pbio.3002720)
Supplement: Attachment — Submitted filename: Yanagi et al.pdf [file pbio.3002720.s007.pdf]

I am reviewing Yanagi et al. In this study they perform a mutagenesis screen to identify modifiers of NGLY1 deficiency in the worm. They identified multiple suppressor modifiers, involved in nucleotide availability, that act independent of NRF1 but still rescue the proteasome defects in NGLY1 deficiency. Overall the study is interesting and adds new insight to the field, but the manuscript is missing a number of key data. It seemed to be a bit cherry picked in what they present. Including these missing data is important.

1. You sequenced 60 suppressor strains. Did you find other suppressors besides the 3 reported genes? If you did, a table of those results would be good.

2. Please provide a multiple species alignment for ent-4.

3. Figures S1 A-C would be nice as part of Figure 1. It's helpful to easily see what types of mutations you are identifying.

4. Figure S1 G should also be in figure 1

5. In lines 230-233 and Figure 2C, why do you not test tald-1 as well? It's odd and a bit glaring to leave that data out. Especially when experiments report directly before and after include tald-1. Please include this data.

6. In lines 240-241 and Figure 2D, why did you not test rsks-1? Again, it's odd when experiments directly before and after include rsks-1. Please include this data.

7. In lines 243-248 and Figure 2E, it is worth a sentence or two of explanation of why you need a rescue of lethality with skn-1 RNAi in the rsks-1/png-1 animals. I know it is addressed in the figure legend, but an assessable description (to a non worm biologist) in the results would help.

8. Line 257-258 needs a reference in regards to the lifespan extension of AMPK in rsks-1 animals.

9. Lines 332-336 and Figure 3E, why were rsks-1 and tald-1 not tested with this specific Aβ experiment. It seems that simple lifespan extension is not the same as life span extension in the presence of Aβ. If it's already been tested for these 2 genes, then explicitly say it here. If it has not been tested. Please provide the data.

10. Lines 341-375 and Figures 4A-C, Why not also test tald-1 and rsks-1 in these intestinal experiments. I don't see why these other two genes wouldn't also function through the intestine or affect nucleotide transport in the intestine.

11. Line 372-374 and Figure 4D. I would not describe the pept-1 effect as no effect. It clearly looks like there is a mild rescue, albeit not statistically significant. It reaches almost 50% with some experiments.

12. Figure 4D-E – it’s odd that the data looks very bimodal in these experiments – 25% difference in some cases. Was this done in two replicates? Two different days? Two blocks? Was this effect corrected for in the statistic?

13. Lines 492-510 – This mTOR hypothesis seems easily testable. The authors should consider some genetic experiments or rapamycin based experiments to address this. This would help add some more depth to the results.

14. There have been a number of transcriptomic studies, modifier studies, and drug screens for NGLY1. Please include some discussion as to why you are identifying these modifiers now and weren’t identified previously. There doesn’t appear to be much hint for a role of nutrients in previous studies.
