# Supplementary material for: Mutations in nucleotide metabolism genes bypass proteasome defects in png-1/NGLY1-deficient Caenorhabditis elegans
Source: PLoS Biol. 2024 Jul 11;22(7):e3002720. doi: 10.1371/journal.pbio.3002720 (PMC11265709; doi:10.1371/journal.pbio.3002720)
Supplement: Attachment — Submitted filename: Comments on-PBIOLOGY-D-23-03351R1.docx [file pbio.3002720.s006.docx]

Comments on “Nucleotide metabolism mutations bypass proteasome defects in png-1/NGLY1 deficient Caenorhabditis elegans” by Yanagi et al.

This manuscript describes a suppressor screening for the hypersensitivity toward proteasome inhibitor, bortezomib. They successfully identified mutations in *rsks-1*, *tald-1*, and *ent-4*. They showed that the suppression is not through SKN-1, and provided convincing evidence to show that, while restriction of nucleotide availability is beneficial to mitigate proteasome insufficiency, a nucleotide-rich diet resulted in exacerbation of the phenotype.

This manuscript is, in general, very well written and easy to follow, and proposes a novel concept that modulation of nucleotide availability could be a therapeutic strategy for NGLY1 deficiency or other diseases associated with compromised proteostasis, which will surely attract the wide audience of this journal. I would thus believe that, after the following points are properly revised, this manuscript will be suitable for publication in *PLOS Biology*.

1	Page 5, lines 107-108; NGLY1 deficiency does not lead to signs of increased accumulation of misfolded proteins or activation of ER stress responses,

- I am just wondering whether the authors also want to mention about the following article, which indicates that many ER stress related genes are upregulated and proteostat-positive aggregates are formed in neurons derived from NGLY1-deficient iPSCs (Manole, et al., Cell Reports 2023).

2.	Page 5, lines 114-116; Indeed, the neurodegenerative consequences of NGLY1 inactivation in mouse and rat brains and those reported following brain-specific inactivation of Nrf1 are similar.

- I am a bit puzzled by this statement as Asahina et al. rather suggested that there is no obvious changes in the level of Nrf1 and their downstream proteins (ex. proteasome subunits). Probably the authors may want to cite papers of brain-specific KO mice and specifically mention which phenotypes the authors want to refer to.

3.	Page 6, lines 162-163. In each case, the mutants show delayed growth under standard culture conditions.

- Can the authors show this data quantitatively? I also wonder how the growth are affected for each double mutant tested. Do they show inverse relationship between growth defect and resistance against BTZ?

4.	Page 7, lines 196-199; However, BTZ survival of png1Δ animals harboring two suppressor mutations --- are not sufficient to completely restore normal resilience to BTZ-induced lethality to png-1Δ animals (Fig. 1e).

- I wonder whether the triple mutant (tald-1 ent-4 rsks-1) showed even greater effect than, for example, double mutant (ex rsks-1 tald-1). LD50 value could be evaluated. I am just curious if there is any specific reason why the authors are not testing the triple mutant at all.

5.	Page 9, lines 257-258; Thus, although AAK-2/AMPK is required for the extended lifespan of rsks-1 animals,

- I am wondering whether this statement needs a reference(s). Is this a well-known fact? Sorry if I miss something.

6.	Fig. 4D; are pho-1 and ent-4 really in the same pathway? If so, can we expect that they do not have an additive effect? The same discussion can be applied for gspd-1 and tald-4, just to further validate the authors’ hypothesis.

Minor points:

Line 177: a re -> are

Line 510: (Fig. 1d, e) -> Can this be Fig. 1b-e? I thought the authors are mentioning the effect of ent-4 mutation (thus comparing Fig. 1b/c with Fig. 1d/e).

Line 636: png-1Δ -> should be italicized (*png-1Δ*) ?

Line 708: in two fragments -> they want to put “:” here (in two fragments:) ?

Lines 757-759: It may be worthwhile to indicate the dilution of each antibody used.

Statistics in Figures: It may be better to use same symbols for statistical analysis; Can the authors use the consistent symbols (**** p<0.0001, *** p<0.001, ** p<0.01, * p<0.05, ns p>0.05) throughout the figure? For example, ** p<0.01 in Fig. 2C, E; in Fig. 3D, it says *** p<0.0001 but it should read p<0.001. Please check.

Figure 1: Why different alleles of ent-4 were used in Fig. 1b, c and d? Are they expected to give essentially same consequence? I am just curious.

Figure 3C: Can the authors indicate the size of molecular weight markers? I am also wondering whether it is worthwhile checking the level of GFP by western blotting, just to confirm the consistency between the level of fluorescence.
