# Supplementary material for: Mutations in nucleotide metabolism genes bypass proteasome defects in png-1/NGLY1-deficient Caenorhabditis elegans
Source: PLoS Biol. 2024 Jul 11;22(7):e3002720. doi: 10.1371/journal.pbio.3002720 (PMC11265709; doi:10.1371/journal.pbio.3002720)
Supplement: Attachment — Submitted filename: Yanagi_Response to reviewers.docx [file pbio.3002720.s008.docx]

Reviewers' comments

**Rev. 1: Tadashi Suzuki - note that this reviewer has signed his review
Comments on “Nucleotide metabolism mutations bypass proteasome defects in png-1/NGLY1 deficient Caenorhabditis elegans” by Yanagi et al.

This manuscript describes a suppressor screening for the hypersensitivity toward proteasome inhibitor, bortezomib. They successfully identified mutations in rsks-1, tald-1, and ent-4. They showed that the suppression is not through SKN-1, and provided convincing evidence to show that, while restriction of nucleotide availability is beneficial to mitigate proteasome insufficiency, a nucleotide-rich diet resulted in exacerbation of the phenotype.

This manuscript is, in general, very well written and easy to follow, and proposes a novel concept that modulation of nucleotide availability could be a therapeutic strategy for NGLY1 deficiency or other diseases associated with compromised proteostasis, which will surely attract the wide audience of this journal. I would thus believe that, after the following points are properly revised, this manuscript will be suitable for publication in PLOS Biology.**

We thank Dr. Suzuki for his positive assessment of the suitability of our work for publication in PLoS Biology and for the multiple insightful and constructive comments.

**1. Page 5, lines 107-108; NGLY1 deficiency does not lead to signs of increased accumulation of misfolded proteins or activation of ER stress responses,

-> I am just wondering whether the authors also want to mention about the following article, which indicates that many ER stress related genes are upregulated and proteostat-positive aggregates are formed in neurons derived from NGLY1-deficient iPSCs (Manole, et al., Cell Reports 2023).**

We thank the reviewer for pointing out this error. We have corrected the manuscript to cite the work of Manole and colleagues. We have also revised the wording of this section to more precisely describe the evidence regarding misfolded protein accumulation and aggregation in specific NGLY1 deficiency models.

**2. Page 5, lines 114-116; Indeed, the neurodegenerative consequences of NGLY1 inactivation in mouse and rat brains and those reported following brain-specific inactivation of Nrf1 are similar.

-> I am a bit puzzled by this statement as Asahina et al. rather suggested that there is no obvious changes in the level of Nrf1 and their downstream proteins (ex. proteasome subunits). Probably the authors may want to cite papers of brain-specific KO mice and specifically mention which phenotypes the authors want to refer to.**

We thank the reviewer for pointing out this error. We did not write this section clearly and failed to cite the relevant literature. We have reorganized this section, and we hope that our reasoning is now clear. We have altered the discussion of mouse phenotypes to emphasize evidence for neurodegenerative phenotypes accompanied by accumulation of ubiquitinated proteins in the brains of both Nrf1 and NGLY1 KO mice. We also refer to the interesting work by Asahina and colleagues demonstrating similar phenotypes despite no changes in Nrf1 or proteasome subunit levels in the rat. We now cite the appropriate work for each of these points.

**3. Page 6, lines 162-163. In each case, the mutants show delayed growth under standard culture conditions.

-> Can the authors show this data quantitatively? I also wonder how the growth are affected for each double mutant tested. Do they show inverse relationship between growth defect and resistance against BTZ?**

We have not quantified the growth rate of the double mutants. Because we observe that the suppressor mutations increase the survival of adult animals exposed to BTZ, our data suggests that the suppressors’ effects are not specific to developmental progression. We therefore think it is unlikely that changing growth rate plays a causal role in resistance to BTZ. We agree that detailed phenotypic characterization of the suppressor mutants, including quantification of growth rate (or other aspects of development) would be interesting, but we think it is outside the scope of this work and would not alter our overall conclusions. However, we do think it is important to show in figure 1A (albeit non-quantitively), the comparison between *png-1∆* and double mutants’ growth on control and BTZ-supplemented plates, since our original mutagenesis screen used developmental progression as a read-out for BTZ sensitivity of the *png-1∆* animals.

**4. Page 7, lines 196-199; However, BTZ survival of png1Δ animals harboring two suppressor mutations --- are not sufficient to completely restore normal resilience to BTZ-induced lethality to png-1Δ animals (Fig. 1e).

-> I wonder whether the triple mutant (tald-1 ent-4 rsks-1) showed even greater effect than, for example, double mutant (ex rsks-1 tald-1). LD50 value could be evaluated. I am just curious if there is any specific reason why the authors are not testing the triple mutant at all.**

We have not constructed the triple mutant. We were motivated to analyze the phenotypes of the double mutant combinations since wanted to test for epistasis patterns that would suggest that some of the suppressors may act together in a linear pathway. We predict (based on the enhanced resistance of each double mutant combination) that the triple mutant would show even further enhanced BTZ resistance. We agree that it would be interesting to see how resistant these animals could get, but this experiment would not provide additional mechanistic insight beyond what we learned from analyzing the double mutants. We realize our statement “… indicating that the suppressors, even in combination, are not sufficient …“ is confusing since we have not tested the effect of all three mutations combined. We have edited this sentence to clarify that we are referring only to the pairwise combinations.

**5. Page 9, lines 257-258; Thus, although AAK-2/AMPK is required for the extended lifespan of rsks-1 animals,

-> I am wondering whether this statement needs a reference(s). Is this a well-known fact? Sorry if I miss something.**

We apologize for the omission; we have added the reference.

**6. Fig. 4D; are pho-1 and ent-4 really in the same pathway? If so, can we expect that they do not have an additive effect? The same discussion can be applied for gspd-1 and tald-4, just to further validate the authors’ hypothesis.**

It is unclear whether *pho-1* and *ent-4* are in the same pathway, since the precise substrates of PHO-1 are unknown. Although it would be interesting to construct a *pho-1; ent-4* double mutant we anticipate that this would be quite difficult. *pho-1* mutant animals display a highly penetrant maternal-effect embryonic lethality/arrest phenotype, which has been attributed to defective maternal acquisition and provision of nutrients that are essential for embryogenesis (Fukushige at al., 2005, PMID: 15733671). We suspected that this defect could be exacerbated by the additional disruption of *ent-4*, so we did not attempt to construct a double mutant. We note that even if *ent-4* and *pho-1* do not act in the same pathway, this would not contradict our hypothesis that dietarily acquired nucleosides/nucleotides can impact proteostasis but could suggest that other PHO-1-dependent nutrients also have an effect.

In regard to genetic analysis of *gspd-1* and *tald-*1, this would be an interesting experiment and could further our understanding of how the pentose phosphate pathway (PPP) functions in *C. elegans*, but might not be highly informative in regards to the connection between the PPP and proteostasis/BTZ resistance. The PPP is reported to be essential in *C. elegans* and many animals, so it is perplexing that our *tald-1* mutants are viable and fertile (even if they are slow growing). Although we have characterized *tald-1* mutants harboring putative null alleles, we cannot completely exclude the possibility that these mutations are not true nulls, or that another enzyme may ‘moonlight’ and allow a low level of PPP function in the absence of TALD-1. The *gsdp-1* mutant that we used in these experiments is a deletion and likely null that causes sterility, so we analyzed (sterile) homozygous mutant animals derived from a heterozygous parent. As a result, we cannot exclude that maternally provided GSPD-1 supports minimal PPP function in these animals. Given these concerns that neither the *tald-1* or *gspd-1* mutants have completely inactivated the PPP, any additive or non-additive effects seen in double mutants would be difficult to interpret.

**Minor points:
Line 177: a re -> are**

Corrected

**Line 510: (Fig. 1d, e) -> Can this be Fig. 1b-e? I thought the authors are mentioning the effect of ent-4 mutation (thus comparing Fig. 1b/c with Fig. 1d/e).**

Corrected. In the revised figure we now refer to Fig. 1e-i.

**Line 636: png-1Δ -> should be italicized (png-1Δ) ?**

Corrected

**Line 708: in two fragments -> they want to put “:” here (in two fragments:) ?**

Corrected.

**Lines 757-759: It may be worthwhile to indicate the dilution of each antibody used.**

Corrected

**Statistics in Figures: It may be better to use same symbols for statistical analysis; Can the authors use the consistent symbols (**** p<0.0001, *** p<0.001, ** p<0.01, * p<0.05, ns p>0.05) throughout the figure? For example, ** p<0.01 in Fig. 2C, E; in Fig. 3D, it says *** p<0.0001 but it should read p<0.001. Please check.**

We thank the reviewer for drawing our attention to the multiple typos we had made in preparing the figure legends. We use the same symbols in all figures (**** p<0.0001, *** p<0.001, ** p<0.01, * p<0.05, ns p>0.05). We have corrected the figure legends to reflect this.

**Figure 1: Why different alleles of ent-4 were used in Fig. 1b, c and d? Are they expected to give essentially same consequence? I am just curious.**

Yes, the different alleles of *ent-4* used in these figure panels (now figures 1e, g and h) are all putative null alleles (either causing a deletion or introducing a premature stop codon, as shown in figure 1b). All are expected to have the same molecular consequence – complete loss of ENT-4 protein. We have confirmed that the alleles cause similar a phenotype in respect of BTZ sensitivity (for example the effect of *ent-4*(*nic738*) shown in figure 1e comparable to the effect of *ent-4*(*ok2161*) shown in figure 1f). We admit that it was not ideal perform the genetic interaction analysis in figures 1h and 1i using multiple *ent-4* alleles. However, given their similar molecular consequences, and the strong genetic interactions we report, this does not undermine our conclusions regarding the genetic relationship between *ent-4* and the other suppressors.

**Figure 3C: Can the authors indicate the size of molecular weight markers? I am also wondering whether it is worthwhile checking the level of GFP by western blotting, just to confirm the consistency between the level of fluorescence.**

We have added labels to show the size of the molecular weight markers in figure 3C.

We have not performed WB to measure GFP levels since we can reliably quantify GFP levels by live imaging. Repeating all experiments with large numbers of synchronized animals (as would be required to generate protein lysates for WB) would be prohibitively time consuming, especially as some of the mutants are slow growing and difficult to maintain in synchronized culture.

**Rev. 2:
I am reviewing Yanagi et al. In this study they perform a mutagenesis screen to identify modifiers of NGLY1 deficiency in the worm. They identified multiple suppressor modifiers, involved in nucleotide availability, that act independent of NRF1 but still rescue the proteasome defects in NGLY1 deficiency. Overall, the study is interesting and adds new insight to the field, but the manuscript is missing a number of key data. It seemed to be a bit cherry picked in what they present. Including these missing data is important.**

We thank the reviewer for their interest in our work. We appreciate that our paper does not include some specific experiments as outlined in reviewer’s comments, and we apologize that the reasons for these missing data were not made clear. In each case, the missing experiments were not conducted for specific practical and/or theoretical reasons. We have outlined these reasons below and updated the manuscript to clarify the reasons why certain data are not included.

**1. You sequenced 60 suppressor strains. Did you find other suppressors besides the 3 reported genes? If you did, a table of those results would be good.**

Unfortunately, we have not identified any other suppressors besides the three reported genes.

**2. Please provide a multiple species alignment for ent-4.**

We aligned ENT-4 to human SLC29A3 and fly ENT2. This multiple species alignment is shown in figure S1D.

**3. Figures S1 A-C would be nice as part of Figure 1. It’s helpful to easily see what types of mutations you are identifying.**

We have added the gene schematics from figure S1 A-C to Figure 1, as panels B-D.

**4. Figure S1 G should also be in figure 1**

We have added Figure S1G to Figure 1, as panel F.

**5. In lines 230-233 and Figure 2C, why do you not test tald-1 as well? It’s odd and a bit glaring to leave that data out. Especially when experiments report directly before and after include tald-1. Please include this data.**

Our initial attempts to generate a *skn-1a tald-1* double mutant were unsuccessful because the two genes are linked on chromosome IV (separated by ~1.8 cM on the genetic map). We had planned to return to this experiment by using CRISPR to generate a new *tald-1* mutation linked *skn-1a*(*mg570*). However, in the meantime we completed the RNAi experiment shown in figure 2E, which demonstrates categorically that the effect of *tald-1* is independent of both SKN-1A and SKN-1C, since both isoforms are depleted by the RNAi. Given that these data already strongly support our conclusion, we did not make a second attempt to construct a *skn-1a tald-1* double mutant. We have explained the reason for this missing experiment in the revised manuscript.

**6. In lines 240-241 and Figure 2D, why did you not test rsks-1? Again, it’s odd when experiments directly before and after include rsks-1. Please include this data.**

Our attempts to cross *rsks-1* with the *gst-4_p_::gfp* reporter were not successful because the reporter is integrated on chromosome III, the same chromosome as the *rsks-1* locus. We presume that the integration site of *gst-4_p_::gfp* is too closely linked to *rsks-1* to isolate a recombinant. We have explained this reason for not analyzing the effect of *rsks-1* in the revised manuscript. Importantly, the effect of *rsks-1*(*RNAi*) on *gst-4_p_::gfp* expression has already been described (Wang et al, 2010, PMID:20700440). We also apologize that in our initial manuscript we failed to reference the follow up work from the Blackwell lab that confirmed that inhibition of mTOR signaling causes SKN-1 activation (Robida-Stubbs et al 2012, PMID: 22560223). We have corrected this omission. Given the strong evidence that *rsks-1* and mTOR signaling influences SKN-1 activity, we do not think it is necessary to include an analysis of the effect of *rsks-1* on *gst-4p::gfp* in our manuscript.

**7. In lines 243-248 and Figure 2E, it is worth a sentence or two of explanation of why you need a rescue of lethality with skn-1 RNAi in the rsks-1/png-1 animals. I know it is addressed in the figure legend, but an assessable description (to a non worm biologist) in the results would help.**

We have added a description and explanation of this effect to the main text, similar to the one included in the figure legend.

**8. Line 257-258 needs a reference in regards to the lifespan extension of AMPK in rsks-1 animals.**

Thank you for alerting us to this omission, we have added the reference.

**9. Lines 332-336 and Figure 3E, why were rsks-1 and tald-1 not tested with this specific Aβ experiment. It seems that simple lifespan extension is not the same as life span extension in the presence of Aβ. If it’s already been tested for these 2 genes, then explicitly say it here. If it has not been tested. Please provide the data.**

We thank the reviewer for this comment and apologize that we did not clearly explain our motivations for only examining the effect of *ent-4* on Aβ paralysis. We have re-written this section to clarify the goal of this experiment.

Because previous work had linked inactivation of *rsks-1* and *tald-1* to enhanced proteostasis, we decided not to test their roles in proteostasis any further. Instead, we chose to focus on the role of *ent-4*, because inactivation of *ent-4* had not been previously linked to changes in proteostasis. There are many assays that could provide insight into altered proteostasis in the *ent-4* mutants, but we chose the amyloid beta assay since it is sensitive, rapid, and our prior data had shown that inactivation of SKN-1A/PNG-1 signaling increases amyloid beta accumulation and toxicity (Lehrbach and Ruvkun, 2019 PMID: 30973820).

We are not aware of any published results showing that *rsks-1* or *tald-1* inactivation have any effect on the accumulation or toxicity of Aβ in *C. elegans*. Although some studies in mouse models have linked mTOR inhibition by rapamycin to reduced amyloid beta accumulation and a reduction in associated pathology (Majumder et al, 2011 PMID: 21980451 and Spilman et al, 2010 PMID: 20376313). Further investigation of proteostasis effects of *rsks-1* and *tald-1* would be interesting, but we do not believe that carrying out these experiments would alter the overall conclusions of our work, since there is already published evidence enhanced proteostasis in each case.

We agree that simple lifespan extension is not the same as the extension of lifespan in the presence of Aβ. Accordingly, we have removed the reference to “lifespan” in this section since our experiments do not directly address the role of any of the suppressors in longevity or lifespan extension, but rather examine specific examples of age-dependent changes in tissue homeostasis and proteostasis.

**10. Lines 341-375 and Figures 4A-C, Why not also test tald-1 and rsks-1 in these intestinal experiments. I don’t see why these other two genes wouldn’t also function through the intestine or affect nucleotide transport in the intestine.**

Our motivation to study the tissue and subcellular site of action of ENT-4 was driven by our surprise in identifying a putative nucleoside transporter in our screen, and our desire to better understand how inactivating this protein could impact BTZ resistance. Before our work ENT-4 had not been studied, so the only information indicating its’ anatomical expression pattern was from single cell RNAseq and tissue-specific transcriptomics, and there was no information on its subcellular localization. Thus, we designed these experiments with the specific intent to better understand the role of ENT-4.

We agree it would be very interesting to discover the tissue(s) of action of TALD-1 and RSKS-1 in controlling BTZ resistance, and to test whether either of these factors play any role in nucleotide transport or its regulation. However, our results showing the ENT-4 functions in the intestine do not necessarily imply that TALD-1 or RSKS-1 should function in the intestine, or that these proteins are involved in nucleotide transport. Moreover, establishing the tissues in which these factors are required are not necessary to support our key finding that modulating nucleotide availability influences proteasome function in *png-1∆* animals. We agree that a more detailed understanding of the relationships between these other suppressors, nucleoside/nucleotide uptake, synthesis, and/or transport between tissues would be of great interest, but it is not necessary to support this main conclusion.

**11. Line 372-374 and Figure 4D. I would not describe the pept-1 effect as no effect. It clearly looks like there is a mild rescue, albeit not statistically significant. It reaches almost 50% with some experiments.**

We have altered this section to reflect the possibility that *pept-1* has a mild but not statistically significant effect on BTZ sensitivity and made a more qualified conclusion. Instead of “increased resistance to BTZ is not a general consequence of impaired nutrient uptake” we now state “increased resistance to BTZ is unlikely to be a general consequence of impaired nutrient uptake”.

**12. Figure 4D-E – it’s odd that the data looks very bimodal in these experiments – 25% difference in some cases. Was this done in two replicates? Two different days? Two blocks? Was this effect corrected for in the statistic?**

These experiments were performed in several independent replicates over a long period spanning approximately 2 years. BTZ-supplemented plates were freshly prepared for each separate replicate, and for each replicate we randomly selected L4 animals from non-synchronized cultures that had not experienced crowding or starvation for at least 2 generations. We agree that these is clear variability in the results, particularly with some of the mutants, and in some cases the distribution of the results appears bimodal. In the table below we summarize the replicate data and show the dates when each replicate experiment was performed. There is no relationship that we can discern between the timing of data collection and the apparent bimodal distribution of the results, which suggests to us that there is no systematic ‘batch effect’ that could alter the interpretation of these data. Our statistical test (Ordinary one-way ANOVA with Dunnett’s multiple comparisons test) does not include a correction for batch effects, as we do not believe this is necessary for the analysis.

| **data collected for Figure 4D** | | |  |
| --- | --- | --- | --- |
| date | genotype | | |
|  | wild type | pho‑1  (ca101ca102) | pept‑1  (lg601) |
| 12/12/20 | 16.7 | 83.3 | 20.0 |
| 12/22/20 | 16.7 | 56.7 | 20.0 |
| 12/25/20 | 10.0 | 83.3 | 43.3 |
| 1/8/21 | 3.3 | 56.7 | 23.3 |
| 1/15/21 | 23.3 |  | 43.3 |

| **data collected for Figure 4E** | | |  |  |  |  |
| --- | --- | --- | --- | --- | --- | --- |
| date | genotype | | | | | |
|  | wild type | gspd‑1  (tm3862) | prps‑1  (gk3067) | pyr‑1  (ok2391) | pyr‑1  (cu8) | umps‑1  (mn160) |
| 11/6/18 | 16.7 |  | 84.0 |  |  | 63.3 |
| 11/13/18 | 30.0 |  | 93.3 |  |  | 33.3 |
| 11/17/18 | 17.9 |  | 66.7 |  |  |  |
| 1/29/19 | 20.0 | 66.7 | 90.0 | 75.0 | 46.7 | 63.3 |
| 2/12/19 | 13.3 | 66.7 | 96.7 | 50.0 | 50.0 | 33.3 |
| 8/14/20 | 23.3 | 66.7 | 90.0 | 75.0 | 46.7 | 36.7 |
| 8/17/20 | 6.7 |  | 73.3 | 53.3 | 70.0 | 36.7 |
| 8/18/20 | 20.0 |  | 96.7 | 50.0 | 50.0 | 33.3 |
| Note: WT data collected in 2018 (gray) is not displayed in figure 4E and was not included in statistical analysis. | | | | | | |

**13. Lines 492-510 – This mTOR hypothesis seems easily testable. The authors should consider some genetic experiments or rapamycin based experiments to address this. This would help add some more depth to the results.**

We appreciate this excellent suggestion. Although this is an exciting future avenue for this work, we believe it is outside the scope of this study.

**14. There have been a number of transcriptomic studies, modifier studies, and drug screens for NGLY1. Please include some discussion as to why you are identifying these modifiers now and weren’t identified previously. There doesn’t appear to be much hint for a role of nutrients in previous studies.**

We thank the reviewer for raising this important point, which we now address in the first paragraph of the revised discussion. Interestingly, although previous studies have not implicated the nucleoside/nucleotide content of the diet, studies in Drosophila and mice do suggest that the diet modulates NGLY1 deficiency phenotypes. We now mention and cite these studies. It is difficult to understand why these different studies have identified diverse dietary modifiers, so we have refrained from speculating on this topic.
